# Associations between Consumption of Dietary Fibers and the Risk of Type 2 Diabetes, Hypertension, Obesity, Cardiovascular Diseases, and Mortality in Chinese Adults: Longitudinal Analyses from the China Health and Nutrition Survey

**DOI:** 10.3390/nu14132650

**Published:** 2022-06-27

**Authors:** Zhaoxia Zhang, Bo Chen, Jingjing Zeng, Menglin Fan, Wenlei Xu, Xiaying Li, Ying Xing, Shaoyong Xu

**Affiliations:** 1 Center for Clinical Evidence-Based and Translational Medicine, Xiangyang Central Hospital, Affiliated Hospital of Hubei University of Arts and Science, Xiangyang 441021, China; zhaoxia19890306@163.com (Z.Z.); chenbofhc@163.com (B.C.); zengjj9@mail2.sysu.edu.cn (J.Z.); xu2972047610@163.com (W.X.); 2 Department of Endocrinology, Daxing Hospital, Xi’an 710000, China; 3 School of Public Health, Tongji Medical College, Huazhong University of Science and Technology, Wuhan 430000, China; fanmenglin08@163.com; 4 College of Medicine, Wuhan University of Science and Technology, Wuhan 430065, China; lixiaying0808@163.com; 5 Department of Endocrinology, Xiangyang Central Hospital, Affiliated Hospital of Hubei University of Arts and Science, Xiangyang 441021, China

**Keywords:** dietary fiber, China nutrition and health database, chronic non-communicable diseases, population-based cohort study

## Abstract

Although many studies have explored the relationship between total dietary fiber intake and the risk of chronic non-communicable diseases, the results are mixed. There is also a lack of research on the association between dietary fiber intake from different food sources and disease. Using data from the China Nutrition and Health Database from 2004 to 2015, Cox proportional risk models were used to explore the associations between total dietary fiber and fiber intake from different food sources and the occurrence of type 2 diabetes, hypertension, obesity, cardiovascular disease, and all-cause mortality. After multi-factorial adjustment, the hazard ratios (95% confidence interval) of total dietary fiber intake (quartile 4 vs. quartile 1) in type 2 diabetes, hypertension, obesity, cardiovascular disease, and all-cause mortality cohorts were 1.20 (0.93, 1.55), 0.91 (0.75, 1.12), 0.93 (0.64, 1.35), 1.13 (0.60, 2.12), 1.13 (0.60, 2.12), and 1.13 (0.84, 1.52). Whole-grain fiber intake was positively associated with hypertension but not with the occurrence of other diseases. No association was observed between legume fibers, fruit fibers, and vegetable fibers in the cohorts of type 2 diabetes, hypertension, obesity, cardiovascular diseases and all-cause mortality. Our study did not find any association between total dietary fiber and dietary fiber intake from different food sources and type 2 diabetes, obesity, cardiovascular disease, and all-cause mortality in the Chinese population. The role of dietary fiber in the Chinese population may be overestimated. More extraordinary efforts are needed to further confirm the association between dietary fiber and these diseases in the Chinese population.

## 1. Introduction

Chronic non-communicable diseases such as diabetes, hypertension, obesity, and cardiovascular disease (CVD) have high incidence and mortality rates worldwide, with type 2 diabetes (T2D) and cardiovascular disease accounting for 11.3% and 30% of total deaths worldwide, respectively [1,2]. The incidence of adult obesity is about 20% [3]. More than 1.39 billion people suffer from hypertension [4], and the public health risks caused by these diseases should not be disregarded.

Many experts have focused their research on preventing the onset of chronic diseases. Dietary fiber consumption has been linked to the prevention of various chronic diseases [5,6,7,8,9]. Dietary fiber is a type of nutrient that is difficult to digest and absorb in the small intestine, and it has several potential mechanisms for preventing chronic diseases, including (1) increasing satiety and possibly promoting weight loss; (2) encouraging intestinal microorganisms to produce short-chain fatty acids with immunomodulatory and anti-inflammatory properties; and (3) experiments in animal models have shown that dietary fiber intake is associated with lower levels of inflammatory and oxidative stress markers [10,11,12]. However, with the continuous refinement and classification of the concept of dietary fiber, studies have found that dietary fiber from different food sources is not all negatively associated with the development of disease [5,7,8,13].

Although more studies have explored the relationship between total dietary fiber intake and the risk of T2D, hypertension, obesity, cardiovascular disease, and all-cause mortality, the results are mixed [5,6,8,14,15,16,17,18]. There is a lack of research on the association between dietary fiber intake from different food sources and disease, especially hypertension, and only a few studies focus on all-cause mortality [9,13,14]. Furthermore, while T2D and cardiovascular disease research are becoming more common, the results from studies of fiber from various dietary sources are also inconsistent [5,7,13]. More importantly, no research has examined the association between dietary fiber intake from various food sources and chronic disease in the Chinese population. Therefore, considering the heterogeneity found in the total dietary fiber intake and dietary fiber intake from different food sources in different populations and its effects on the health statuses, these associations may require further analysis in Chinese populations that traditionally consume diets rich in plant foods.

This study aimed to determine the association between fiber consumption from various dietary sources and T2D, hypertension, obesity, cardiovascular disease, and all-cause mortality in the Chinese population.

## 2. Materials and Methods

### 2.1. Study Design and Participants

Data from the China Nutrition and Health Database (CHNS) were used in this study, a multi-purpose longitudinal open cohort study that began in 1989 and was followed up in 1991, 1993, 1997, 2000, 2004, 2006, 2009, 2011, and 2015. CHNS used a multi-stage stratified whole-group random sampling strategy to sample people from 15 provinces with different populations, geography, economy and public resources in the eastern, central and western regions of China. Demographic, socioeconomic, lifestyle, dietary, and health data is collected during each survey wave. Blood samples were collected and analyzed during the 2009 CHNS. The scientific rationale and design of the CHNS were previously reported [19,20]. The survey was conducted according to the guidelines of the Declaration of Helsinki and was approved by the Institutional Review Committees of the University of North Carolina at Chapel Hill (UNC-CH) and the National Institute for Nutrition and Health, Chinese Center for Disease Control and Prevention; each participant provided informed consent.

Because new food codes were implemented in 2004, CHNS data from 2004 to 2015 were used in this analysis. We first excluded participants who were under 18 years old at baseline, participants with abnormal dietary energy data (e.g., a daily energy intake <800 kcal or >4200 kcal for men and <600 kcal or >3500 kcal for women), and participants with a history of cardiovascular disease, cancer, death, or pregnancy at baseline. The initial wave of these surveys served as a baseline. Finally, the final analysis comprised a total of 9376 participants.

### 2.2. Dietary Assessment

Details on dietary measures are available elsewhere [21]. In brief, at the time of the baseline (2004 or 2006) survey, qualified investigators obtained individual dietary data through face-to-face interviews in each survey round. Individual diets were evaluated several times by recalling the food consumed by individuals during three 24-hour periods. Participants were asked to list all foods and beverages consumed during 24 h, with the types and amounts of items documented based on food models and images, supported by standard sizes (household containers, grams indicated on the packaging). Three consecutive days (2 weekdays and 1 weekend day) were assigned at random during the week, and each sampling unit was nearly balanced across the seven days of the week. The Chinese Food Composition Table (2004 edition) was used to compute dietary component intakes (energy, protein, fat, carbohydrate, dietary fiber), and food groups such as cereals, legumes, vegetables, and fruits were also categorized using the Chinese Food Composition Table. This study did not cover supplement consumption.

To ensure the stability of the dietary data, we used average year data for 2004 and 2006 as the baseline dietary assessment. When participants participated in only one of the survey years, the current year’s dietary intake could be utilized as the baseline assessment. This research method can also be seen in other articles [13,22].

### 2.3. Measurement

Questionnaires were used to collect demographics and lifestyle information, such as age, gender, cigarette and alcohol use, education, physical activity, place of residence, and area. Physical activity was estimated as the duration of total physical activity and reported as the metabolic equivalent task (MET)-minutes/week. Height and weight were assessed using conventional protocols and calibrated equipment by certified medical practitioners. Body mass index (BMI) was calculated as weight (kg)/squared height (m^2^). Professional researchers estimated blood pressure as the mean of three independent measurements.

### 2.4. Case Ascertainment

T2D. A questionnaire-based interview was used to establish diabetes status at each follow-up appointment. Blood samples were also taken and examined in 2009. T2D was characterized as meeting at least one of the following criteria in the 2009 study, according to the American Diabetes Association diagnostic criteria: (1) fasting blood glucose concentration ≥ 7.0 mmol/L (126 mg/dL); (2) HbA1c ≥ 6.5%; (3) self-reported T2D diagnosis or use of hypoglycemic medicine. T2D was defined as self-reported diabetes or use of hypoglycemic medication in 2015. Previous research demonstrated that self-reported diabetes is a relatively useful approach for determining the diabetes status of Chinese study participants [23].

Hypertension. At 10 min of rest, a standard mercury sphygmomanometer was used to monitor diastolic and systolic blood pressures on subjects’ left or right arms, and the mean readings were measured three times and recorded. Hypertension was defined as (1) a systolic blood pressure ≥140 mm Hg; (2) a diastolic blood pressure ≥90 mm Hg; or (3) self-reporting of a diagnosis of hypertension or currently on oral anti-hypertensive medication during follow-up.

Obesity. Each participant’s weight and height were measured by a trained health worker. BMI ≥ 28.0 kg/m^2^ was considered obese according to the Expert Consensus on Weight Management Process for Overweight or Obese People (2021).

Cardiovascular disease. Myocardial infarction or stroke was used to characterize the cardiovascular disease. The following questions were used to determine myocardial infarction and stroke information: “Has your doctor ever told you that you have a myocardial infarction?”. “Have you been diagnosed with stroke by your doctor?” cardiovascular disease was defined as answering yes to any of these questions.

All-cause mortality. The census confirmed participants’ death status based on information submitted in each survey wave and the household system. The year of death was recorded if the participant died.

### 2.5. Statistical Analysis

Descriptive analyses were reported as the mean ± standard deviation (SD) or median (interquartile range) for continuous variables and frequency (percentage) for categorical variables. ANOVA, Kruskal–Wallis test or chi-square test showed statistical differences between quartiles.

We used Cox proportional risk models to assess the association between total dietary fiber and fiber intake from different food sources and the occurrence of type 2 diabetes, hypertension, obesity, cardiovascular disease, and all-cause mortality. The time indicator was the follow-up time from 2004 to the disease onset or cut-off date. To test for potential nonlinear associations, we tested for linear trends using the median score per quantile. All statistical tests were two-sided and performed using SAS 9.4 (SAS Institute, Cary, NC, USA). *p* < 0.05 was considered statistically significant.

## 3. Results

### 3.1. Description of the Study Population

**Figure 1:**
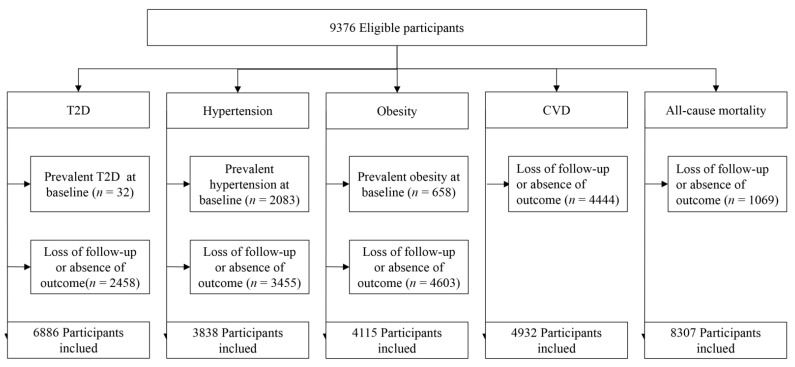
Participants flowchart.

For each particular disease, 9376 participants were eligible; the baseline data excluded cases with existing disease and cases with lost follow-up or missing outcomes, as shown in Figure 1. A total of 6886 cases in the T2D cohort, 3838 cases in the hypertension cohort, 4115 cases in the obesity cohort, 4932 cases in the cardiovascular disease cohort, and 8307 cases in the all-cause mortality cohort were finally included. The T2D cohort of participants was divided into quartiles based on dietary fiber intake, and their baseline characteristics are shown in Table 1. Notably, only 25% of participants had a daily dietary fiber intake of more than 13.5 g/day, which is well below the 25–30 g/day recommended in the Chinese Dietary Guidelines 2022 [24]. Baseline characteristics of hypertension, obesity, cardiovascular disease, and all-cause mortality cohorts are shown in Tables S1–S4 in the Supplementary Materials.

### 3.2. Associations between Total Dietary Fiber Intake and T2D, Hypertension, Obesity, CVD, and All-Cause Mortality

Among the 6886 participants with T2D followed up, 650 cases developed T2D, and the incidence density (1000 person-years) of T2D by quartiles of total dietary fiber intake was 7.81, 9.28, 9.38, and 9.61 from quartile 1 (Q1) to quartile 4 (Q4), respectively, with a hazard ratio (HR)_Q4 vs. Q1_ and 95% confidence interval (95% CI) of 1.22 (0.97, 1.53), *p* > 0.05. Among the 3838 participants with hypertension at follow-up, a total of 1178 developed hypertension, and the incidence density (1000 person-years) of hypertension by quartiles of total dietary fiber intake was 29.21, 29.35, 28.78, and 28.81 for Q1–Q4. HR_Q4 vs. Q1_ was 0.99 (0.84, 1.17), *p* > 0.05. In the follow-up cohort of 4115 cases with obesity, 379 cases developed obesity, and the incidence density (1000 person-years) of obesity in Q1–Q4 was 7.43, 8.67, 9.01, and 9.23, and HR_Q4 vs. Q1_ was 1.11 (95% CI: 0.82, 1.50), *p* > 0.05. In the follow-up cohort of 4932 cases with cardiovascular disease cohort, a total of 127 cases developed cardiovascular disease, and the incidence density (1000 person-years) of cardiovascular disease in Q1–Q4 was 1.79, 2.47, 2.48, and 2.23, respectively; HR_Q4 vs. Q1_ was 1.25 (95% CI: 0.73, 2.15), *p* > 0.05. In the followed-up cohort of 8307 all-cause deaths, a total of 468 cases of mortality occurred, and the incidence density (1000 person-years) of mortality from Q1–Q4, it was 6.16, 4.68, 4.45, and 5.72, respectively, with an HR_Q4 vs. Q1_ of 0.93 (95% CI: 0.73, 1.18), *p* > 0.05.

**Figure 2:**
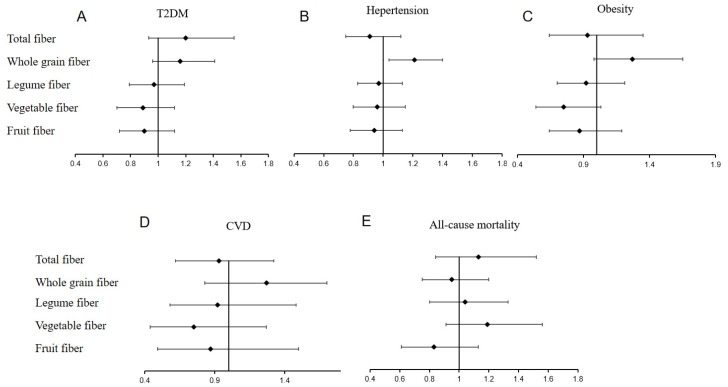
Associations between the consumption of dietary fibers from different sources and (**A**) T2DM, (**B**) hypertension, (**C**) obesity, (**D**) CVD, (**E**) mortality from all causes.

After multi-factorial adjustment, HR_Q4 vs. Q1_ was 1.20 (95% CI: 0.93,1.55), 0.91 (95% CI: 0.75, 1.12), 0.93 (95% CI: 0.64, 1.35), 1.13 (95% CI: 0.60, 2.12), and 1.13 (95%CI: 0.84, 1.52) in the cohorts with diabetes, hypertension, obesity, cardiovascular disease, and all-cause mortality, respectively; *p*-trend > 0.05 (see Table 2, Figure 2).

### 3.3. Associations between Whole-Grain Fiber Intake and T2D, Hypertension, Obesity, CVD, and All-Cause Mortality

The whole-grain fiber intake was divided into terciles (T1–T3). In the cohort of T2D, hypertension, obesity, CVD and all-cause mortality, the HR_T3 vs.__T1_ were 1.31 (95% CI: 1.09, 1.57), 1.29 (95% CI: 1.13, 1.47), 1.62 (95% CI: 1.28, 2.05), 1.43 (95% CI: 0.97, 2.12), and 0.87 (95% CI: 0.69, 1.08), *p* > 0.05. With the addition of fruit fiber, vegetable fiber, and legume fiber on the basis of the adjustment for multiple factors of total dietary fiber, HR_T3 vs.__T1_ in the cohorts of T2D, hypertension, obesity, CVD, and all-cause mortality were 1.16 (95% CI: 0.96, 1.41), 1.21 (95% CI: 1.04, 1.40), 1.27 (95% CI: 0.98, 1.65), 1.29 (95% CI: 0.85, 1.96), and 0.95 (95% CI: 0.75, 1.20), *p* > 0.05 (Table 3, Figure 2). The results show that whole-grain fiber is positively associated with the incidence of T2D, hypertension, and obesity in the unadjusted model and not with the incidence of cardiovascular disease and all-cause mortality. After multi-factorial adjustment, whole-grain fiber intake was positively associated with hypertension but not with the onset of other diseases.

### 3.4. Associations between Legume Fiber Intake and T2D, Hypertension, Obesity, CVD, and All-Cause Mortality

In the terciles of legume fiber intake, The HR_T3 vs__.__T1_ in the cohorts of T2D, hypertension, obesity, CVD, and all-cause mortality were 1.12 (95% CI: 0.93, 1.33), 1.07 (95% CI: 0.94, 1.22), 1.06 (95%CI: 0.84, 1.33), 1.06 (95%CI: 0.70, 1.60), and 0.86 (95%CI: 0.69, 1.07), *p* > 0.05. After adding fruit fiber, vegetable fiber, and whole-grain fiber to adjust for the multiple factors of total dietary fiber, HR_T3 vs__.__T1_ in T2D, hypertension, obesity, CVD and all-cause mortality cohorts were 0.97 (95% CI: 0.79, 1.19), 0.97 (95% CI: 0.83, 1.13), 0.92 (95% CI: 0.70, 1.21), 0.90 (95% CI: 0.56, 1.46), and 1.04 (95% CI: 0.80, 1.33), *p* > 0.05 (Table 4, Figure 2). The results show that legume fiber was not associated with the development of T2D, hypertension, obesity, CVD and all-cause mortality.

### 3.5. Associations between Vegetable Fiber Intake and T2D, Hypertension, Obesity, CVD, and All-Cause Mortality

After vegetable fiber intake by quartiles classification, HR_Q4 vs__. Q1_ in the cohorts of T2D, hypertension, obesity, CVD, and all-cause mortality were 0.85 (95% CI: 0.68, 1.06), 0.90 (95% CI: 0.77, 1.06), 0.72 (95% CI: 0.54, 0.96), 0.85 (95% CI: 0.53, 1.36), and 1.14 (95% CI: 0.89, 1.46), respectively, *p* > 0.05. After adding fruit fiber, legume fiber, and whole-grain fiber to adjust for multiple factors of total dietary fiber, HR_Q3 vs__. Q1_ in the cohorts of T2D, hypertension, obesity, CVD and all-cause mortality were 0.89 (95% CI: 0.70, 1.12), 0.96 (95% CI: 0.80, 1.15), 0.75 (95% CI: 0.54, 1.03), 0.77 (95% CI: 0.46, 1.29), 1.19 (95% CI: 0.91, 1.56), respectively, *p* > 0.05 (Table 5, Figure 2). The results show that vegetable fiber was not associated with the onset of T2D, hypertension, obesity, CVD and all-cause mortality.

### 3.6. Associations between Fruit Fiber Intake and T2D, Hypertension, Obesity, CVD, and All-Cause Mortality

Because the number of people who did not eat fruit fiber was more than half, the fruit fiber intake was divided into the fruit fiber intake group and the non-fruit fiber intake group. Compared to the non-fruit fiber intake group, the HR in the intake group in the cohorts of T2D, hypertension, obesity, CVD, and all-cause mortality were 0.98 (95% CI: 0.80, 1.20), 0.86 (95% CI: 0.73, 1.02), 1.01 (95% CI: 0.77, 1.33), 0.89 (95%CI: 0.55, 1.46), and 0.58 (95%CI: 0.44, 0.77). After adding the factors of legume fiber, vegetable fiber, and whole-grain fiber to adjust for the multiple factors of total dietary fiber, the HR of the intake group were 0.90 (95% CI: 0.72, 1.12), 0.94 (95% CI: 0.78, 1.13), 0.87 (95% CI: 0.64, 1.19), 0.91 (95% CI: 0.53, 1.54), 0.83 (95% CI: 0.61, 1.13) (Table 6, Figure 2). The results show that fruit fiber was negatively associated with the onset of all-cause mortality in the unadjusted model but not with other diseases. However, after adding vegetable fiber, legume fiber, and whole-grain fiber to adjust for multiple factors of total dietary fiber, fruit fiber was not associated with the onset of T2D, hypertension, obesity, CVD, and all-cause mortality.

## 4. Discussion

In a large prospective cohort of Chinese adults, we concluded that total dietary fiber and dietary fiber intake from different food sources were not significantly associated with chronic diseases, such as T2D, hypertension, obesity, cardiovascular disease, and all-cause mortality.

There are inconsistent findings from previous studies on dietary fiber intake and the incidence of type 2 diabetes, hypertension, obesity, cardiovascular disease, and all-cause mortality. On the relationship between dietary fiber intake and the risk of developing type 2 diabetes, a meta-analysis conducted in 2015 [5], which included 18 cohort studies, suggested that dietary fiber intake was associated with a lower risk of diabetes (HR: 0.82; 95% CI: 0.69, 0.97) but was no longer statistically significant after adjusting for BMI. However, a study in Japan in (2021) [25] and a study in France in (2020) [13] suggested a negative association between fiber intake and T2D. On the relationship between dietary fiber intake and the risk of developing hypertension, two earlier studies showed that fiber intake was not associated with developing hypertension [26,27]. However, a 2021 US study [28] suggested dietary fiber was independently associated with a reduced risk of diastolic hypertension (OR = 0.848, 95% CI 0.770, 0.934) and systolic hypertension (OR = 0.906, 95% CI 0.826, 0.993) after adjustments were made for confounding factors. On the relationship between dietary fiber intake and the risk of cardiovascular disease, a 2016 Iranian study [7] and a 2022 study from NHNES [29] suggested a negative association between fiber intake and the development of cardiovascular disease, but a 2020 French study [13] concluded that dietary fiber intake was not associated with the development of cardiovascular disease (OR = 0.86, 95% CI: 0.70, 1.06). On the relationship between dietary fiber intake and the risk of obesity development, a 2010 European study [8] suggested that total fiber intake was negatively associated with increased body weight and waist circumference. For total fiber intake above 10 g/day, the combined estimated change in body weight was −39 g/year (95% CI: −71, −7), and the change in waist circumference was −0.08 cm/year (95% CI: −0.11, −0.05). Studies on fiber intake and all-cause mortality yielded inconsistent findings, such as a meta-analysis in 2015 [30], and a 2020 Japanese study [17], which suggested a negative association between dietary fiber intake and all-cause mortality. However, a 2020 French cohort study [13] suggested that dietary fiber was not associated with all-cause mortality (OR = 0.98, 95% CI: 0.72, 1.33).

Our results only partially reproduce some of the findings of previous studies, which may be related to demographic differences (e.g., country, race, age, sex), and differences in dietary patterns. In addition, the dietary fiber intake in our population is relatively low and may be lower than an intake that would provide significant health benefits. For example, a meta-analysis that included 13 prospective studies [31] and a French cohort study [6] showed that when the total dietary fiber intake was higher than 25 g/day, it was negatively associated with developing T2D and hypertension. In contrast, the highest quartile of dietary fiber intake in our study population was >13.5 g/day, which is much lower than the dietary fiber intake in previous study populations, which may be one of the reasons why our study yielded no association.

Ample evidence on dietary fiber suggests that whole-grain fiber may be more likely than other fibers to reduce the risk of developing diabetes and obesity [8,32,33,34,35,36]. Many studies suggested that the beneficial effects of whole-grain fiber may be due to the fiber co-intake of other nutrients (e.g., magnesium and vitamins B1, C, and E), while the lower glycemic index of higher whole-grain fiber diets may have reduced the risk of diabetes and obesity development [35]. However, whole-grain fiber was not associated with either diabetes or obesity in our study. This result may be due to our study population’s relatively low intake of whole-grain fiber, with the Chinese population generally consuming refined grains with shallow fiber content. Some studies reported that the effect of cereal fiber on reducing the risk of developing diabetes and obesity is mainly related to the intake of whole-grains [11], while other characteristics of refined grains, such as the nature of their high glycemic index, may influence the observed results.

Some studies reported that high dietary fiber consumption decreased the risk of hypertension or BP [37,38,39], although other research studies reported that fiber intake was not significantly associated with hypertension [40,41]. Compared with the lowest tercile, the HR (95% CI) of hypertension for the highest tercile intakes of cereal fiber was 0.80 (0.67, 0.96) in US research [37]. The data from SWAN Study suggest that dietary fiber intake, especially from grains, contributes to a lower risk of systolic and diastolic BP in middle-aged women [28]. However, our study shows that whole-grain fiber is positively associated with the incidence of hypertension, which is inconsistent with previous findings. Differences in results may be related to sample size, ethnicity, dietary patterns, and the environment of the study population. More research is needed to confirm this result.

Some prospective cohort studies suggest that vegetable and fruit fibers may reduce the risk of cardiovascular disease more than other fibers [7,14]. It has been proposed that vegetable and fruit fibers, due to their higher content of soluble and insoluble fibers, can reduce the activity of fibrinogen activator inhibitor type 1 and coagulation factor VII [42,43,44,45] and affect gut microbiota, modifying the inflammatory response of the body [46]. These may be some of the mechanisms through which they reduce the risk of cardiovascular disease. However, our study did not yield relevant conclusions, which may be influenced by the method of investigation, and differences in the amount and type of fruits and vegetables consumed in different seasons may impact our findings.

Prospective studies in the United States and Europe have shown that cereal fiber intake is significantly associated with a lower total mortality [47,48]. In contrast, studies from Japan concluded that cereal fiber intake was not associated with mortality, but fiber intake in legumes, vegetables, and fruits was significantly and negatively associated with total mortality [17]. However, our study concluded that neither total dietary fiber nor fiber intake from various food sources was significantly associated with mortality. Several studies have reached different conclusions, with genetic differences among countries and races and differences in dietary habits contributing to the final results, in addition to the low dietary fiber intake of our study population.

Studies on dietary fiber and chronic diseases such as hypertension, obesity, cardiovascular disease, and all-cause mortality in the Chinese population are lacking. A 2021 study [49] involving 3250 middle-aged and elderly participants in Hangzhou reported that dietary fiber intake was associated with a reduced risk of newly diagnosed T2D (odds ratio (OR) = 0.70, 95% CI: 0.49, 1.0), and another study [50] reported that dietary fiber intake was associated with a reduced risk of prediabetes in a population in Tianjin (OR = 0.85, 95% CI: 0.75, 0.98). We found that the highest quantile of dietary fiber intake in both study populations (>15.1 g and >21.4 g, respectively) was higher than in our study population (>13.5 g). This further confirms that the dietary fiber intake of our study population is too small to reduce the risk of disease. Of course, the region, age, gender, and dietary habits of the study population also contribute to a different fiber intake.

The strength of our study is it’s prospective study, the large sample size, and the systematic exploration of the relationship between dietary fiber intake and various chronic diseases and all-cause mortality in the Chinese population. Another major strength of our study is the detailed collection of dietary intakes, collected through repeatedly validated 24-hour dietary records based on an extensive database of more than 6900 food items. This allowed us to examine the associations between different food groups and various chronic diseases [51]. However, some limitations should also be acknowledged. Firstly, limited by the database, although our model adjusted for various potential confounders, residual confounding by the family history of the disease, certain medical conditions and drugs variables, or metabolic factors may persist. Secondly, due to the limitations of the cohort study itself, dietary intake may be influenced by economic and social development, which may affect the associations with disease. However, to ensure the stability of the dietary data, we used average year data for 2004 and 2006 as the baseline dietary assessment. Lastly, in our study, disease detection was primarily based on self-report, and despite this relatively valid method, misclassification bias could not be ruled out.

## 5. Conclusions

Our study did not find an association between total dietary fiber and dietary fiber intake from various food sources with type 2 diabetes, hypertension, obesity, cardiovascular disease, and all-cause mortality in the Chinese population. However, our study shows that whole-grain fiber is positively associated with the incidence of hypertension. The role of dietary fiber in the Chinese population may be overestimated. To further confirm the association between dietary fiber and these diseases in the Chinese population, more extraordinary efforts are needed in the future to increase the intake of dietary fiber in the Chinese population and to try to diversify the food groups in the dietary pattern (whole-grains, legumes, vegetables, fruits, and meat).

## Figures and Tables

**Table 1:** Characteristics of the study population at inclusion in the T2D cohort, according to quartiles of total dietary fiber intakes.

Characteristics	Fiber Intake (g/d)				*p*
	Quartile 1 (<6.21)	Quartile 2 (6.21–8.81)	Quartile 3 (8.81–13.50)	Quartile 4 (>13.50)	
Number	1611	1718	1799	1778	
Age (years)	47.62 ± 14.89	46.93 ± 13.71	47.44 ± 13.41	47.77 ± 13.34	0.307
Male, n (%)	667 (42.14)	797 (46.69)	865 (49.01)	931 (52.57)	<0.001
BMI (kg/m^2^)	22.97 ± 3.33	23.04 ± 3.41	23.25 ± 3.28	23.10 ± 3.29	0.097
Waist circumference (cm)	80.04 ± 9.98	80.44 ± 9.85	81.34 ± 9.78	81.32 ± 9.58	0.001
Systolic blood pressure (mmHg)	122.46 ± 17.89	121.43 ± 17.35	121.97 ± 18.70	121.04 ± 17.29	0.136
Diastolic blood pressure (mmHg)	79.24 ± 10.79	78.58 ± 10.47	78.57 ± 11.94	78.11 ± 10.92	0.048
Physical activity (MET-MIN/day)	274.28 (0.00–1531.29)	978.96 (0.00–1992.86)	993.01 (0.00–2090.14)	1079.04 (0.00–2220.01)	<0.001
Smoking, n (%)					
Yes	461 (29.21)	555 (32.44)	613 (34.61)	628 (35.44)	0.006
No	1117 (70.79)	1156 (67.56)	1158 (65.39)	1144 (64.56)	
Alcohol drinking, n (%)					
Yes	444 (28.14)	555 (32.44)	638 (36.09)	649 (36.60)	<0.001
No	1134 (71.86	1156 (67.56)	1130 (63.91)	1124 (63.40)	
Educational level, n (%)					0.004
Primary school or below	699 (44.16)	768 (44.99)	835 (47.34)	916 (51.72)	
Junior high school	497 (31.40)	558 (32.69)	571 (32.37)	515 (29.08)	
Senior high school	237 (14.97)	229 (13.42)	214 (12.13)	211 (11.91)	
College and above	150 (9.48)	152 (8.90)	144 (8.16)	129 (7.28)	
Regions, n (%)					
Urban	600 (37.24)	535 (31.14)	531 (29.85)	444 (24.97)	<0.001
Rural	1011 (62.76)	1183 (68.86)	1248 (70.15)	1334 (75.03)	
Total energy intake (kcal/day)	1869.11 ± 547.14	2115.88 ± 550.74	2280.64 ± 576.40	2479.94 ± 598.07	<0.001
Total carbohydrate intake (g/d)	263.19 ± 79.91	311.06 ± 85.94	338.77 ± 100.03	378.01 ± 108.77	<0.001
Total protein intake (g/d)	55.26 ± 18.97	62.11 ± 19.08	69.03 ± 20.59	76.46 ± 25.01	<0.001
Total fatty intake (g/d)	63.99 ± 35.09	66.95 ± 35.87	68.67 ± 35.74	70.90 ± 37.45	<0.001
Na intake (mg/d)	60.91 (14.41–485.21)	52.81 (16.20–829.61)	60.60 (16.63–1130.85)	49.61 (16.74–850.52)	<0.001
Whole-grain fiber intake (g/d)	0.37 ± 0.79	0.72 ± 1.26	1.18 ± 1.99	2.58 ± 5.49	<0.001
Legume fiber intake (g/d)	0.11 ± 0.32	0.33 ± 0.74	0.76 ± 1.53	2.56 ± 5.52	<0.001
Vegetable fiber intake (g/d)	2.01 ± 0.96	3.37 ± 1.32	4.82 ± 2.36	10.86 ± 9.53	<0.001
Fruit fiber intake (g/d)	0.10 ± 0.33	0.21 ± 0.59	0.37 ± 0.93	0.48 ± 1.37	<0.001

Descriptive analyses of continuous variables were conducted by means ± standard deviations (SD) or medians (interquartile range), and categorical variables were described by number (percentage). Analysis of variance or Kruskal–Wallis test was used for continuous variables, and chi-square test was used for categorical variables. BMI: body mass index.

**Table 2:** Multi-variable adjusted HRs (95% CI) of T2D, hypertension, obesity, CVD, and all-cause mortality according to quartiles of total dietary fiber.

Total Fiber Intake					
	Quartile 1	Quartile 2	Quartile 3	Quartile 4	*p*-Trend
T2D					
Number of cases	132	166	174	178	
Person-years	16,902	17,888	18,545	18,515	
Incidence density (1000 person-years)	7.81	9.28	9.38	9.61	
T2D ^a^	1.0	1.18 (0.94, 1.48)	1.19 (0.95, 1.50)	1.22 (0.97, 1.53)	0.12
T2D ^b^	1.0	1.12 (0.88, 1.43)	1.13 (0.88, 1.44)	1.20 (0.93, 1.55)	0.08
Hypertension					
Number of cases	270	297	307	304	
Person-years	9241	10,116	10,667	10,552	
Incidence density (1000 person-years)	29.21	29.35	28.78	28.81	
Hypertension ^a^	1.0	1.01 (0.86, 1.19)	0.99 (0.84, 1.17)	0.99 (0.84, 1.17)	0.63
Hypertension ^c^	1.0	0.98 (0.82, 1.18)	0.86 (0.71, 1.04)	0.91 (0.75, 1.12)	0.25
Obesity					
Number of cases	75	98	109	97	
Person-years	10,087	11,308	12,089	11,781	
Incidence density (1000 person-years)	7.43	8.67	9.01	8.23	
Obesity ^a^	1.0	1.17 (0.86, 1.57)	1.21 (0.90, 1.63)	1.11 (0.82, 1.50)	0.14
Obesity ^c^	1.0	1.09 (0.78, 1.52)	1.02 (0.73, 1.44)	0.93 (0.64, 1.35)	0.40
CVD					
Number of cases	22	38	35	32	
Person-years	12,276	15,363	14,124	14,289	
Incidence density (1000 person-years)	1.79	2.47	2.48	2.23	
CVD ^a^	1.0	1.56 (0.93, 2.64)	1.38 (0.81, 2.36)	1.25 (0.73, 2.15)	0.34
CVD ^c^	1.0	1.56 (0.90, 2.70)	1.24 (0.70, 2.23)	1.13 (0.60, 2.12)	0.07
All-cause mortality					
Number of cases	133	105	101	129	
Person-years	21,571	22,398	22,663	22,543	
Incidence density (1000 person-year)	6.16	4.68	4.45	5.72	
All-cause mortality ^a^	1.0	0.76 (0.59, 0.98)	0.72 (0.56, 0.94)	0.93 (0.73, 1.18)	0.11
All-cause mortality ^c^	1.0	1.05 (0.79, 1.38)	0.95 (0.70, 1.27)	1.13 (0.84, 1.52)	0.14

HRs were examined using Cox proportional hazard models. ^a^: Confounding factors were not adjusted. ^b^: Adjusted for age, sex, BMI, education, regions, physical activity, smoking status, alcohol drinking, total energy intake, total carbohydrate intake, protein intake, and fatty intake. ^c^: Adjusted for age, sex, BMI, education, regions, physical activity, smoking status, alcohol drinking, total energy intake, total carbohydrate intake, protein intake, fatty intake, systolic blood pressure, diastolic blood pressure, and Na intake.

**Table 3:** Multi-variable adjusted HRs (95%CI) of T2D, hypertension, obesity, CVD, and all-cause mortality according to terciles of whole-grain fiber.

Whole-Grain Fiber				
	Tercile 1	Tercile 2	Tercile 3	*p*-Trend
T2D ^a^	1.00	1.33 (1.11, 1.62)	1.31 (1.09, 1.57)	0.06
T2D ^b1^	1.00	1.16 (0.94, 1.43)	1.16 (0.96, 1.41)	0.12
Hypertension ^a^	1.00	1.13 (0.96, 1.31)	1.29 (1.13, 1.47)	0.04
Hypertension ^c1^	1.00	1.05 (0.88, 1.25)	1.21 (1.04, 1.40)	0.03
Obesity ^a^	1.00	1.67 (1.29, 2.15)	1.62 (1.28, 2.05)	0.73
Obesity ^c1^	1.00	1.23 (0.91, 1.65)	1.27 (0.98, 1.65)	0.55
CVD ^a^	1.00	1.04 (0.64, 1.70)	1.43 (0.97, 2.12)	0.16
CVD ^c1^	1.00	0.94 (0.56, 1.59)	1.29 (0.85, 1.96)	0.52
All-cause mortality ^a^	1.00	0.83 (0.65, 1.06)	0.87 (0.69, 1.08)	0.14
All-cause mortality ^c1^	1.00	0.98 (0.75, 1.28)	0.95 (0.75, 1.20)	0.09

^a^: Confounding factors were not adjusted. ^b1^: HRs were examined using Cox proportional hazard models. adjusted for age, sex, BMI, education, regions physical activity, smoking status, alcohol drinking, total energy intake, total carbohydrate intake, protein intake, fatty intake, legume fiber, fruit fiber, and vegetable fiber. ^c1^: HRs were examined using Cox proportional hazard models adjusted for age, sex, BMI, education, regions, physical activity, smoking status, alcohol drinking, total energy intake, total carbohydrate intake, protein intake, fatty intake, systolic blood pressure, diastolic blood pressure, Na intake, legume fiber, fruit fiber, and vegetable fiber.

**Table 4:** Multi-variable adjusted HR (95% CI) of T2D, hypertension, obesity, CVD, and all-cause mortality according to terciles of legume fiber.

Legume Fiber				
	Tercile 1	Tercile 2	Tercile 3	*p*-Trend
T2D ^a^	1.00	1.15 (0.92, 1.43)	1.12 (0.93, 1.33)	0.43
T2D ^b2^	1.00	1.09 (0.87, 1.38)	0.97 (0.79, 1.19)	0.24
Hypertension ^a^	1.00	0.95 (0.79, 1.15)	1.07 (0.94, 1.22)	0.37
Hypertension ^c2^	1.00	1.01 (0.82, 1.24)	0.97 (0.83, 1.13)	0.16
Obesity ^a^	1.00	0.84 (0.59, 1.20)	1.06 (0.84, 1.33)	0.20
Obesity ^c2^	1.00	0.84 (0.57, 1.25)	0.92 (0.70, 1.21)	0.08
CVD ^a^	1.00	1.06 (0.63, 1.76)	1.06 (0.70, 1.60)	0.12
CVD ^c2^	1.00	1.09 (0.64, 1.85)	0.90 (0.56, 1.46)	0.67
All-cause mortality ^a^	1.00	0.91 (0.70, 1.20)	0.86 (0.69, 1.07)	0.19
All-cause mortality ^c2^	1.00	1.07 (0.81, 1.42)	1.04 (0.80, 1.33)	0.46

^a^: Confounding factors were not adjusted. ^b2^: HRs were examined using Cox proportional hazard models. Adjusted for age, sex, BMI, education, regions physical activity, smoking status, alcohol drinking, total energy intake, total carbohydrate intake, protein intake, fatty intake, whole-grain fiber, fruit fiber, and vegetable fiber. ^c2^: HRs were examined using Cox proportional hazard models. Adjusted for age, sex, BMI, education, regions, physical activity, smoking status, alcohol drinking, total energy intake, total carbohydrate intake, protein intake, fatty intake, systolic blood pressure, diastolic blood pressure, Na intake, whole-grain fiber, fruit fiber, and vegetable fiber.

**Table 5:** Multivariable adjusted HRs (95% CI) of T2D, hypertension, obesity, CVD, and all-cause mortality according to quartiles of vegetable fiber.

Vegetable Fiber					
	Quartile 1	Quartile 2	Quartile 3	Quartile 4	*p*-Trend
T2D ^a^	1.00	0.85 (0.68, 1.06)	0.97 (0.79, 1.20)	0.85 (0.68, 1.06)	0.24
T2D ^b3^	1.00	0.83 (0.66, 1.05)	0.97 (0.77, 1.21)	0.89 (0.70, 1.12)	0.34
Hypertension ^a^	1.00	0.93 (0.79, 1.09)	0.92 (0.78, 1.08)	0.90 (0.77, 1.06)	0.18
Hypertension ^c3^	1.00	1.01 (0.85, 1.20)	0.96 (0.81, 1.15)	0.96 (0.80, 1.15)	0.12
Obesity ^a^	1.00	0.72 (0.54, 0.96)	0.84 (0.64, 1.10)	0.72 (0.54, 0.96)	0.46
Obesity ^c3^	1.00	0.78 (0.57, 1.08)	0.87 (0.64, 1.19)	0.75 (0.54, 1.03)	0.71
CVD ^a^	1.00	0.72 (0.43, 1.18)	0.83 (0.51, 1.33)	0.85 (0.53, 1.36)	0.19
CVD ^c3^	1.00	0.70 (0.41, 1.18)	0.75 (0.45, 1.26)	0.77 (0.46, 1.29)	0.83
All-cause mortality ^a^	1.00	0.97 (0.75, 1.25)	0.88 (0.67, 1.14)	1.14 (0.89, 1.46)	0.16
All-cause mortality ^c3^	1.00	1.01 (0.77, 1.34)	1.14 (0.85, 1.51)	1.19 (0.91, 1.56)	0.47

^a^: Confounding factors were not adjusted. ^b3^: HRs were examined using Cox proportional hazard models. adjusted for age, sex, BMI, education, regions, physical activity, smoking status, alcohol drinking, total energy intake, total carbohydrate intake, protein intake, fatty intake, whole-grain fiber, fruit fiber, and legume fiber. ^c3^: HRs were examined using Cox proportional hazard models. Adjusted for age, sex, BMI, education, regions, physical activity, smoking status, alcohol drinking, total energy intake, total carbohydrate intake, protein intake, fatty intake, systolic blood pressure, diastolic blood pressure, Na intake, whole-grain fiber, fruit fiber, and legume fiber.

**Table 6:** Multivariable adjusted HRs (95%CI) of T2D, hypertension, obesity, CVD, and all-cause mortality according to the classification of intake of fruit fiber or not.

Fruit Fiber		
	No Intake Group	Intake Group
T2D ^a^	1.00	0.98 (0.80, 1.20)
T2D ^b4^	1.00	0.90 (0.72, 1.12)
Hypertension ^a^	1.00	0.86 (0.73, 1.02)
Hypertension ^c4^	1.00	0.94 (0.78, 1.13)
Obesity ^a^	1.00	1.01 (0.77, 1.33)
Obesity ^c4^	1.00	0.87 (0.64, 1.19)
CVD ^a^	1.00	0.89 (0.55, 1.46)
CVD ^c4^	1.00	0.91 (0.53, 1.54)
All-cause mortality ^a^	1.00	0.58 (0.44, 0.77)
All-cause mortality ^c4^	1.00	0.83 (0.61, 1.13)

^a^: Confounding factors were not adjusted. ^b4^: HRs were examined using Cox proportional hazard models. adjusted for age, sex, BMI, education, regions, physical activity, smoking status, alcohol drinking, total energy intake, total carbohydrate intake, protein intake, fatty intake, whole-grain fiber, vegetable fiber, and legume fiber. ^c4^: HRs were examined using Cox proportional hazard models. adjusted for age, sex, BMI, education, regions, physical activity, smoking status, alcohol drinking, total energy intake, total carbohydrate intake, protein intake, fatty intake, systolic blood pressure, diastolic blood pressure, Na intake, whole-grain fiber, vegetable fiber, and legume fiber.

## Data Availability

Data from CHNS described in the manuscript will be made publicly and freely available without restriction at China Health and Nutrition Survey. Available online: https://www.cpc.unc.edu/projects/china/data/datasets/index.html (accessed on 12 May 2022).

## Supplementary Materials

The following supporting information can be downloaded at: https://www.mdpi.com/article/10.3390/nu14132650/s1, Table S1: Characteristics of the study population at inclusion in the hypertension cohort, according to quartiles of total dietary fiber intakes. Table S2: Characteristics of the study population at inclusion in the obesity cohort, according to quartiles of total dietary fiber intakes. Table S3: Characteristics of the study population at inclusion in the CVD cohort, according to quartiles of total dietary fiber intakes. Table S4: Characteristics of the study population at inclusion in the all-cause mortality cohort, according to quartiles of total dietary fiber intakes.
