# Supplementary material for: Associations between Consumption of Dietary Fibers and the Risk of Type 2 Diabetes, Hypertension, Obesity, Cardiovascular Diseases, and Mortality in Chinese Adults: Longitudinal Analyses from the China Health and Nutrition Survey
Source: Nutrients. 2022 Jun 27;14(13):2650. doi: 10.3390/nu14132650 (PMC9268526; doi:10.3390/nu14132650)
Supplement: Supplementary file 1 [file nutrients-14-02650-s001.zip › nutrients-1770211-supplementary.pdf]

Table S1 Characteristics of the study population at inclusion in the hypertension cohort, according to quartiles of total dietary fiber intakes.

Characteristics	Fiber intake（g/d）				*p*
	Quartile 1 (<6.26)	Quartile 2 (6.26-8.84)	Quartile 3 (8.84-13.66)	Quartile 4 (>13.66)	
Number	877	954	1005	1002	
Age (years)	44.17 ± 12.84	44.07 ± 11.90	44.90 ± 12.26	45.35 ± 12.21	0.068
Male, n (%)	364 (42.62)	433 (45.68)	474 (47.49)	507 (50.75)	0.005
BMI (kg/m2)	22.46 ± 2.95	22.53 ± 3.08	22.79 ± 3.01	22.77 ± 3.16	0.045
Waist circumference(cm)	78.14 ± 8.89	78.91 ± 9.08	79.54 ± 8.91	80.05 ± 9.22	<0.001
Systolic blood pressure (mm Hg)	115.12 ± 11.61	115.00 ± 10.53	114.39 ± 11.55	115.00 ± 11.21	0.519
Diastolic blood pressure (mm Hg)	75.19 ± 7.78	75.25 ± 7.51	74.06 ± 8.18	74.66± 7.96	0.005
Physical activity (MET-MIN/day)	587.57 (0.00-1645.71)	1131.43 (0.00-2132.14)	1225.71 (0.00-2255.14)	1325.04 (16.50-2468.57)	<0.001
Smoking, n (%)					
Yes	244 (28.67)	317 (33.37)	343 (34.27)	339 (33.93)	0.041
No	607 (71.33)	633 (66.63)	658 (65.73)	660 (66.07)	
Alcohol drinking, n (%)					
Yes	226 (33.37)	317 (36.87)	368 (36.06)	361 (36.06)	<0.001
No	626 (66.63)	633 (63.13)	630 (63.94)	640 (63.94)	
Educational level, n (%)					
Primary school or below	329 (38.52)	395 (41.67)	455 (45.64)	497 (49.75)	0.001
Junior high school	316 (37.00)	346 (36.50)	358 (35.91)	311 (31.13)	
Senior high school	134 (15.69)	136 (14.35)	112 (11.23)	130 (13.01)	
College and above	75 (8.78)	71 (7.49)	72 (7.22)	61 (6.11)	
Regions, n (%)					
Urban	289 (32.95)	263 (27.57)	265 (26.37)	219 (21.86)	<0.001
Rural	588 (67.05)	691 (72.43)	740 (73.630)	783 (78.14)	
Total energy intake (kcal/day)	1895.94 ± 531.12	2136.44 ± 531.85	2307.63 ± 564.51	2512.07 ± 590.30	<0.001

Total carbohydrate intake (g/d)	272.23 ± 81.08	320.93 ± 83.49	347.35 ± 101.19	386.09 ± 108.81	<0.001
Total protein intake (g/d)	55.69 ± 17.67	62.49 ± 18.48	69.87 ± 19.61	77.59 ± 24.28	<0.001
Total fatty intake (g/d)	63.29 ± 32.71	64.52 ± 33.57	67.41 ± 33.94	70.44 ± 36.98	<0.001
Na intake (mg/d)	189.41 (108.91-343.23)	266.23 (151.96-525.92)	362.54 (172.49-766.27)	375.73 (179.55-799.16)	<0.001
Whole-grain fiber intake (g/d)	0.38 ± 0.80	0.71 ± 1.28	1.18 ± 1.99	2.52 ± 5.70	<0.001
Legume fiber intake (g/d)	0.11 ± 0.31	0.34 ± 0.76	0.77 ± 1.55	2.55 ± 5.57	<0.001
Vegetable fiber intake (g/d)	2.02 ± 0.96	3.38 ± 1.33	4.83 ± 2.37	11.09 ± 9.87	<0.001
Fruit fiber intake (g/d)	0.08 ± 0.31	0.18 ± 0.54	0.37 ± 0.93	0.47 ± 1.37	<0.001

Descriptive analyses of continuous variables were conducted by means ± standard deviations (SD) or medians (interquartile range), and categorical variables were described by number (percentage). Analysis of variance or Kruskal-Wallis test was used for continuous variables, and chi square test was used for categorical variables. BMI: body mass index.

Table S2 Characteristics of the study population at inclusion in the obesity cohort, according to quartiles of total dietary fiber intakes.

**Characteristics**	**Fiber intake（g/d）**				***p***
	**Quartile 1 (<6.20)**	**Quartile 2 (6.20-8.82)**	**Quartile 3 (8.82-13.54)**	**Quartile 4 (>13.54)**	
Number	917	1028	1099	1071	
Age (years)	46.75 ± 12.88	46.32 ± 12.29	46.72 ± 12.31	47.31 ± 12.33	0.336
Male, n (%)	372 (41.56)	472 (46.18)	530 (48.53)	538 (50.42)	0.001
BMI (kg/m^2)	22.45 ± 2.63	22.34 ± 2.63	22.66 ± 2.62	22.57 ± 2.59	0.037
Waist circumference (cm)	78.60 ± 9.01	78.71 ± 8.53	79.64 ± 8.69	79.94 ± 8.55	0.001
Systolic blood pressure (mm Hg)	120.50 ± 16.33	119.30 ± 16.02	119.53 ± 17.01	120.41 ± 17.38	0.304
Diastolic blood pressure (mm Hg)	78.41 ± 10.35	77.67 ± 10.25	77.31 ± 11.25	77.66 ± 11.17	0.182
Physical activity (MET-MIN/day)	608.14 (0.00-1645.71)	1024.50 (0.00-2057.14)	1146.64 (0.00-2214.86)	1164.86 (0.00-2331.43)	<0.001
Smoking, n (%)					
Yes	252 (28.25)	354 (34.57)	381 (34.76)	378 (35.36)	0.003
No	640 (71.75)	670 (65.43)	715 (65.24)	691 (64.64)	

Alcohol drinking, n (%)					
Yes	245 (33.98)	348 (33.98)	405 (37.05)	392 (36.67)	<0.001
No	648 (66.02)	676 (66.02)	688 (62.95)	677 (63.33)	
Educational level, n (%)					
Primary school or below	388 (43.35)	463 (45.30)	529 (48.49)	554 (51.92)	0.012
Junior high school	314 (35.08)	345 (33.76)	369 (33.82)	309 (28.96)	
Senior high school	126 (14.08)	142 (13.89)	122 (11.18)	135 (12.65)	
College and above	67 (7.49)	72 (7.05)	71 (6.51)	69 (6.47)	
Regions, n (%)					
Urban	307 (33.48)	283 (27.53)	286 (26.02)	242 (22.60)	<0.001
Rural	610 (66.52)	745（72.47)	813 (73.98)	829 (77.40)	
Total energy intake (kcal/day)	1888.35 ± 529.72	2132.15 ± 538.02	2293.56 ± 572.15	2484.81 ± 595.70	<0.001
Total carbohydrate intake (g/d)	269.64 ± 79.60	317.03 ± 82.71	341.98 ± 100.38	378.20 ± 108.20	<0.001
Total protein intake (g/d)	55.45 ± 18.09	62.33 ± 18.69	69.03 ± 19.89	76.81 ± 25.01	<0.001
Total fatty intake (g/d)	63.45 ± 33.61	65.54 ± 34.84	68.39 ± 34.28	71.11 ± 37.38	<0.001
Na intake (mg/d)	186.78 (107.43-341.19)	266.29 (156.50-513.93)	366.08 (171.43-757.17)	375.46 (177.40-792.19)	<0.001
Whole-grain fiber intake (g/d)	0.37 ± 0.81	0.70 ± 1.26	1.18 ± 2.00	2.52 ± 5.58	<0.001
Legume fiber intake (g/d)	0.11 ± 0.31	0.32 ± 0.75	0.77 ± 1.56	2.56 ± 5.51	<0.001
Vegetable fiber intake (g/d)	1.99 ± 0.96	3.37 ± 1.32	4.83 ± 2.37	11.04 ± 9.62	<0.001
Fruit fiber intake (g/d)	0.08 ± 0.31	0.20 ± 0.58	0.36 ± 0.92	0.46 ± 1.37	<0.001

Descriptive analyses of continuous variables were conducted by means ± standard deviations (SD) or medians (interquartile range), and categorical variables were described by number (percentage). Analysis of variance or Kruskal-Wallis test was used for continuous variables, and chi square test was used for categorical variables. BMI: body mass index.

Table S3 Characteristics of the study population at inclusion in the CVD cohort, according to quartiles of total dietary fiber intakes.

Characteristics	Fiber intake（g/d）				*p*
	Quartile 1 (<6.21)	Quartile 2 (6.21-8.82)	Quartile 3 (8.82-13.53)	Quartile 4 (>13.53)	
Number	1116	1233	1284	1299	
Age (years)	46.68 ± 13.58	46.38 ± 12.62	46.93 ± 12.51	47.16 ± 12.54	0.468
Male, n (%)	458 (41.94)	572 (46.66)	624 (48.90)	670 (51.74)	<0.001
BMI (kg/m2)	22.99 ± 3.33	23.06 ± 3.42	23.19 ± 3.14	23.16 ± 3.34	0.461
Waist circumference (cm)	79.7 ± 9.85	80.41 ± 9.73	80.96 ± 9.51	81.36 ± 9.64	0.001
Systolic blood pressure (mmHg)	121.64 ± 17.42	120.65 ± 16.61	121.11 ± 18.17	121.26 ± 17.56	0.632
Diastolic blood pressure (mmHg)	78.95 ± 10.67	78.60 ± 10.46	78.13 ± 11.72	78.28 ± 11.19	0.323
Physical activity (MET-MIN/day)	477.42 (0.00-1600.50)	1006.29 (0.00-2046.00)	1131.43 (0.00-2183.14)	1164.43 (0.00-2317.71)	<0.001
Smoking, n (%)					
Yes	307 (28.22)	415 (33.77)	449 (35.05)	451 (34.83)	0.002
No	781 (71.78)	814 (66.23)	832 (64.95)	844 (65.17)	
Alcohol drinking, n (%)					
Yes	303 (27.80)	414 (33.69)	476 (37.25)	482 (37.16)	<0.001
No	787 (72.20)	815 (66.31)	802 (62.75)	815 (62.84)	
Educational level, n (%)					
Primary school or below	474 (43.41)	553 (45.11)	615 (48.24)	671 (51.81)	0.003
Junior high school	370 (33.88)	417 (34.01)	429 (33.65)	384 (29.65)	
Senior high school	164 (15.02)	167 (13.62)	145 (11.37)	160 (12.36)	
College and above	84 (7.69)	89 (7.26)	86 (6.75)	80 (6.18)	
Regions, n (%)					
Urban	384 (34.41)	355 (28.79)	343 (26.71)	295 (22.71)	<0.001
Rural	732 (65.59)	878 (71.21)	941 (73.29)	1004 (77.29)	<0.001
Total energy intake (kcal/day)	1870.58 ± 532.14	2117.39 ± 546.16	2291.21 ± 570.91	2501.04 ± 595.38	<0.001

Total carbohydrate intake (g/d)	267.42 ± 79.49	314.35 ± 84.58	341.25 ± 100.99	382.00 ± 108.63	<0.001
Total protein intake (g/d)	55.13 ± 18.03	62.09 ± 18.93	69.17 ± 34.64	77.57 ± 25.34	<0.001
Total fatty intake (g/d)	62.74 ± 33.16	65.46 ± 34.59	68.44 ± 34.64	70.94 ± 37.15	
Na intake (mg/d)	183.47 (107.19-342.09)	267.84 (154.73-521.22)	375.58 (173.26-757.01)	384.31 (183.99-796.31)	<0.001
Whole-grain fiber intake (g/d)	0.37 ± 0.81	0.70 ± 1.26	1.18 ± 2.00	2.52 ± 5.58	<0.001
Legume fiber intake (g/d)	0.11 ± 0.31	0.32 ± 0.75	0.77 ± 1.56	2.56 ± 5.51	<0.001
Vegetable fiber intake (g/d)	1.99 ± 0.96	3.37 ± 1.32	4.83 ± 2.37	11.04 ± 9.62	<0.001
Fruit fiber intake (g/d)	0.08 ± 0.31	0.20 ± 0.58	0.36 ± 0.92	0.46 ± 1.37	<0.001

Descriptive analyses of continuous variables were conducted by means ± standard deviations (SD) or medians (interquartile range), and categorical variables were described by number (percentage). Analysis of variance or Kruskal-Wallis test was used for continuous variables, and chi square test was used for categorical variables. BMI: body mass index.

Table S4 Characteristics of the study population at inclusion in the all-cause mortality cohort, according to quartiles of total dietary fiber intakes.

**Characteristics**	**Fiber intake（g/d）**				***p***
	**Quartile 1 (<6.21)**	**Quartile 2 (6.21-8.81)**	**Quartile 3 (8.81-13.53)**	**Quartile 4 (>13.53)**	
Number	2018	2082	2106	2101	
Age (years)	48.38 ± 15.99	47.05 ± 14.15	47.52 ± 14.11	48.04 ± 13.97	0.019
Male, n (%)	835 (42.15)	975 (47.17)	1045 (49.98)	1096 (52.44)	<0.001
BMI (kg/m^2)	22.96 ± 3.38	23.09 ± 3.43	23.24 ± 3.28	23.14 ± 3.27	0.067
Waist circumference(cm)	80.29 ± 10.17	80.69 ± 9.89	81.50 ± 9.88	81.38 ± 9.52	0.001
Systolic blood pressure (mmHg)	123.06 ± 18.69	121.76 ± 17.69	122.46 ± 19.01	121.77 ± 18.20	0.096
Diastolic blood pressure (mmHg)	79.39 ± 11.05	78.81 ± 10.74	78.90 ± 12.20	78.26 ± 11.06	0.025
Physical activity (MET-MIN/day)	240.00 (0.00-1440.00)	848.57 (0.00-1920.00)	960.00 (0.00-2019.00)	993.00 (0.00-2123.14)	<0.001
Smoking, n (%)					<0.0001
Yes	580 (29.34)	674 (32.56)	731 (34.86)	742 (35.49)	

No	1397 (70.66)	1396 (67.44)	1366 (65.14)	1349 (64.51)	
Alcohol drinking, n (%)					<0.001
Yes	547 (27.70)	677 (32.69)	750 (35.82)	764 (36.52)	
No	1428 (72.30)	1394 (67.31)	1344 (64.18)	1328 (63.48)	
Educational level, n (%)					0.005
Primary school or below	860 (43.41)	895 (43.30)	948 (45.36)	1038 (49.67)	
Junior high school	621 (31.35)	675 (32.66)	675 (32.30)	625 (29.90)	
Senior high school	301 (15.19)	281 (13.59)	261 (12.49)	256 (12.25)	
College and above	199 (10.05)	216 (10.45)	206 (9.86)	171 (8.18)	
Regions, n (%)					<0.001
Urban	813 (40.29)	718 (34.49)	673 (31.96)	580 (27.61)	
Rural	1205 (59.71)	1364 (65.51)	1433 (68.04)	1521 (72.39)	
Total energy intake (kcal/day)	1838.92 ± 554.39	2107.11 ± 552.19	2271.51 ± 584.61	2461.00 ± 612.32	<0.001
Total carbohydrate intake (g/d)	257.22 ± 80.55	307.56 ± 86.11	334.24 ± 99.91	372.66 ± 110.49	<0.001
Total protein intake (g/d)	54.78 ± 19.26	62.44 ± 19.56	68.92 ± 20.82	76.59 ± 25.58	<0.001
Total fatty intake (g/d)	63.69 ± 35.56	67.45 ± 35.91	69.97 ± 36.78	71.18 ± 37.91	<0.001
Na intake (mg/d)	181.35 (101.98-338.71)	271.27 (150.60-516.51)	373.92 (175.57-749.91)	385.27 (183.29-802.64)	<0.001
Whole-grain fiber intake (g/d)	0.37 ± 0.80	0.74 ± 1.26	1.19 ± 2.01	2.57 ± 5.55	<0.001
Legume fiber intake (g/d)	0.11 ± 0.32	0.30 ± 0.74	0.80 ± 1.56	2.56 ± 5.52	<0.001
Vegetable fiber intake (g/d)	2.01 ± 0.96	3.34 ± 1.32	4.85 ± 2.37	11.08 ± 9.62	<0.001
Fruit fiber intake (g/d)	0.07 ± 0.31	0.20 ± 0.59	0.36 ± 0.92	0.44 ± 1.37	<0.001

Descriptive analyses of continuous variables were conducted by means ± standard deviations (SD) or medians (interquartile range), and categorical variables were described by number (percentage). Analysis of variance or Kruskal-Wallis test was used for continuous variables, and chi square test was used for categorical variables. BMI: body mass index.
